# Comorbidity in gout at the time of first diagnosis: sex differences that may have implications for dosing of urate lowering therapy

**DOI:** 10.1186/s13075-018-1596-x

**Published:** 2018-06-01

**Authors:** Panagiota Drivelegka, Valgerdur Sigurdardottir, Anna Svärd, Lennart T. H. Jacobsson, Mats Dehlin

**Affiliations:** 1 Department of Rheumatology and Inflammation Research, Sahlgrenska Academy, University of Gothenburg, Grona Straket 12, Sahlgrenska University Hospital, 413 45 Gothenburg, Sweden; 2 Centre of Clinical Research Dalarna, Falun, Sweden

**Keywords:** Gout, Comorbidity, Epidemiology, Gender, Urate lowering treatment

## Abstract

**Background:**

The aim of this study is to examine the occurrence of comorbidities at the time of first diagnosis of gout compared with matched population controls, overall and by sex, as well as to examine the crude and age-standardized prevalence of these comorbidities in men and women with gout at first diagnosis.

**Methods:**

A population-based study used data from Swedish national and regional registers, including 14,113 gout patients aged ≥ 20 years, with a first recorded diagnosis of gout between 1 January 2006 and 31 December 2012, and 65,782 population controls, matched by age, sex and county. Prevalence ratios (95% confidence intervals) comparing gout cases and controls were calculated, overall and by sex. Crude and age-standardized prevalence (95% confidence interval) of all comorbidities in gout patients were calculated, to show differences between sexes, taking also the higher age at diagnosis in women into account.

**Results:**

All examined comorbidities were 1.2–2.5-fold more common in gout patients at diagnosis than in population controls in both sexes. Women with gout were on average 6 years older than men at first gout diagnosis and most comorbidities, including obesity and diuretic use, were or tended to be more frequent in women than in men. When standardizing for age, women had a higher prevalence of thromboembolism (6.6% vs 5.2%) and chronic obstructive pulmonary disease (3.1% vs 2.4%). Men, on the other hand, had a higher prevalence of coronary heart disease (9.4% vs 6.4%), atrial fibrillation (9.0% vs 6.0%), congestive heart failure (7.7% vs 6.6%) and stroke (4.1% vs 3.3%).

**Conclusions:**

The occurrence of most comorbidities was significantly increased at first diagnosis of gout in both sexes. Women were older at diagnosis and had higher occurrence of most comorbidities, including obesity and diuretic use, factors that increase serum urate, and this needs to be taken into account when starting and optimizing urate lowering therapy. These sex differences were attenuated when standardizing for age and the occurrence of cardiovascular diseases was actually higher in men.

**Electronic supplementary material:**

The online version of this article (10.1186/s13075-018-1596-x) contains supplementary material, which is available to authorized users.

## Background

Gout is the most common form of inflammatory arthritis and is caused by deposition of monosodium urate (MSU) crystals in peripheral joints and surrounding tissues as a result of chronic elevation of serum urate (SU) levels. The incidence and prevalence of gout are increasing in many parts of the world and the prevalence is > 1% in North America and Europe [1].

Gout has been linked with an increased occurrence of several comorbidities, which all may influence the overall effect on quality of life and increased mortality [2–4]. Some of these comorbidities, such as use of diuretics [5, 6], renal disease [7–14], obesity [15, 16], transplantation [17–20], psoriasis [21, 22] and alcohol overconsumption [23, 24], have also been suggested to increase SU levels and hence be part of the causal pathway for developing hyperuricemia and clinical gout. Epidemiological studies examining the relationship between gout and risk for cardiovascular diseases (CVD) overall and in men and women separately report conflicting findings, with a significant association reported by some studies [3, 4, 25, 26], but not others [27–29]. There is only limited evidence for positive associations with cerebrovascular disease [30–33] or peripheral vascular disease (PVD) [34, 35].

Current clinical guidelines and management pathways endorsed by the European League against Rheumatism [36], American College of Rheumatology [37] and British Society for Rheumatology [38] agree on the importance of assessing comorbidities before deciding to initiate treatment of gout with urate lowering therapy (ULT). In addition, there are also results supporting the need for higher doses of ULT if patients have comorbid conditions, such as renal disease, use of diuretics or obesity [39]. This effect of decreased eGFR and diuretic use is probably explained by their ability to increase SU levels and not by any effect on the dose–response relationship between given allopurinol dose and change in urate levels [40].

It is well known that several comorbidities occur more commonly in patients with gout compared to the general population [20, 41–43], although there are relatively few population representative studies from Europe [43]. Furthermore, the timing of comorbidity occurrence relative to gout diagnosis has not always been addressed. In particular, there are very few studies examining the prevalence of comorbidities in patients with gout by sex [44–47] and none of them have presented results at the time of gout diagnosis. If such differences between sexes were found, they could indicate that pathological pathways differ in importance in men and women. In addition, such differences could be of importance to take into consideration when initiating and dosing ULT [39].

Using healthcare register data in the Western Swedish Health Care Region (VEGA) to identify cases and population registers to identify matched controls, and linking these groups to several national registers, this study aimed to: examine the occurrence of several comorbidities at the time of first diagnosis of gout compared with matched controls, overall and by sex; and examine the crude and age-standardized prevalence of these comorbidities in men and women with gout at first diagnosis.

## Methods

### Study design

We conducted a population-based and register-based case–control study using register data from 1 January 2000 through 31 December 2012 comparing patients at the time of first gout diagnosis to matched general population controls.

Ethical approval for the study was granted from the Ethical Review Board of Gothenburg, Sweden. Informed consent from the patients was not needed as the study only involved quality register linkage and no actual handling of patients.

### Setting

According to Statistics Sweden, in 2016 the population of Sweden was 9,995,153 and in Västra Götaland, a county in the western part of Sweden, where the study was conducted, the population was 1,671,783 (http://www.scb.se/hitta-statistik/statistik-efter-amne/befolkning/befolkningens-sammansattning/befolkningsstatistik/).

Swedish health care is public and tax funded. Health and demographic information on all inhabitants is recorded in a series of different healthcare registers. Linkage of data from these registers is possible using the 10-digit personal identification number automatically assigned to all Swedish residents [48].

### Data sources

The Western Swedish Health Care Region (VEGA) was used to identify cases with gout. This register contains information back to 2000 about all healthcare contacts at inpatient and outpatient specialty clinics, as well as at primary care clinics, and includes the date of visit and diagnoses given by the treating physician according to the Swedish version of the International Statistical Classification of Diseases (ICD). Since 1997, the 10th version of ICD (ICD-10) is used in Sweden. The vast majority of patients with gout are treated by general practitioners.

The Swedish Prescribed Drug Register (PDR) (http://www.socialstyrelsen.se/register/halsodataregister/lakemedelsregistret) contains information about all prescribed drugs dispensed by Swedish pharmacies since July 2005. This register was used to determine the exposure of patients and controls to treatment with diuretics within 6 months prior to first diagnosis of gout, as well as to support the obesity and hypertension diagnoses. The Anatomical Therapeutic Chemical Classification System (ATC codes) was used to identify the medical treatments (Additional file 1: Table S1).

Demographic data were obtained from Statistics Sweden (http://www.scb.se/en/), which holds data on immigration, emigration and residency. Data on education level were retrieved from the Longitudinal Integration Database for Health Insurance and Labor Market Studies (LISA), which is administered by Statistics Sweden and holds annual registers on all individuals 16 years of age and older.

Vital status on 31 December 2012 was determined via the Cause-of-death Register (https://www.socialstyrelsen.se/register/dodsorsaksregistret), which provides information on the date and cause(s) of death for all residents since 1961.

### Study populations

**Fig. 1:**
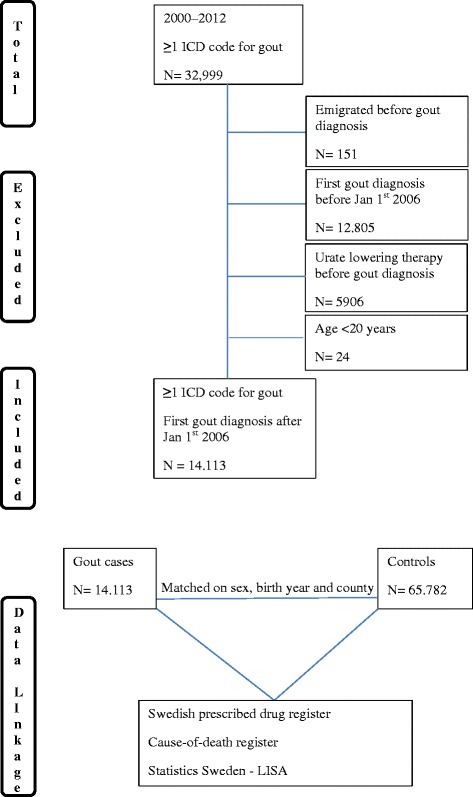
Study design. ICD International Statistical Classification of Diseases, LISA Longitudinal Integration Database for Health Insurance and Labor Market Studies

We identified all patients who attended any inpatient, outpatient or primary care clinic between 1 January 2000 and 31 December 2012. In this study we included all patients who had received their first ICD-coded diagnosis corresponding to gout (Additional file 1: Table S1) between 1 January 2006 and 31 December 2012. This time period was chosen in order to be able to link our data to the PDR and to be able to define these cases as incident. Cases with a diagnosis of gout before that period, cases receiving ULT before gout diagnosis and cases aged < 20 years were excluded (Fig. 1). Up to five general population comparators, alive and without gout by the time of the index patients’ first gout diagnosis were identified for each gout patient and matched by year of birth, sex and county from the population register held by Statistics Sweden.

### Comorbidities and medication of interest

We classified comorbidities into two diagnostic categories: comorbidities which have been ‘suggested to increase the SU level’ and consequently increase the risk for clinical gout (psoriasis, transplantation, renal disease, use of diuretics, obesity and alcoholism); and ‘other comorbidities’, such as diabetes, hypertension, coronary heart disease (CHD), congestive heart failure (CHF), atrial fibrillation (AF), stroke, thromboembolism, PVD and chronic obstructive pulmonary disease (COPD), in which the causal relationship to urate levels and clinical gout is not clear. With the exceptions of hypertension and obesity (based on both ICD codes and/or ever previously dispensed prescription of antihypertensive or anti-obesity drugs respectively according to ATC codes in the PDR), comorbidities were defined as the presence of at least one visit to a physician in primary or specialized care with a corresponding ICD-coded diagnosis (Additional file 1: Table S1). Treatment with diuretics (including thiazide, loop and thiazide-related diuretics) was defined as having dispensed a prescription of these agents within 6 months prior to first diagnosis of gout.

### Comparison by sex

We compared demographic characteristics and pattern of comorbidities separately in men and women, with their respective controls.

### Statistical analysis

Data are expressed as mean ± SD for continuous variables and number and percentages for categorical variables. The prevalence of a specific comorbidity was calculated by dividing the number of subjects diagnosed with that comorbidity prior to the date of first gout diagnosis of the index patient as numerator (for both cases and controls) by the total number of patients with gout or controls as denominator. Prevalence ratios (PRs) and 95% confidence intervals (CIs) were used to estimate the association between gout and each coexisting medical condition. In a sensitivity analysis, only cases with two or more visits with an ICD-coded diagnosis of gout [49] were included and compared with their matched controls. In the sex-stratified analyses we compared both the crude and standardized prevalence for each comorbidity. Standardization was done by the indirect method, with the Swedish census population in 2012 as the standard population. Nonoverlapping 95% CIs were considered statistically significant for all comparisons. All analyses were conducted using SAS software, version 9.3 (SAS Institute Inc. Cary, NC, USA).

## Results

### Population

A total of 14,113 individuals who received their first ICD-coded gout diagnosis between 1 January 2006 and 31 December 2012 were identified and matched to 65,782 general population comparator subjects (Fig. 1). There were 4600 women and 9513 men matched to 22,052 and 43,730 controls, respectively (Table 1). Women were approximately 6 years older than men at the time of gout diagnosis and had lower education level (Table 1).

**Table 1:** Demographic characteristics of the study population and prevalence (95% CI) of comorbidities in gout cases at the time of first diagnosis and statistical comparisons between all gout patients and controls

	Gout cases			Controls			PR (95% CI) (total gout cases – total controls)	*p* value (total gout cases – total controls)
	Men (*N* = 9513)	Women (*N* = 4600)	Total (*N* = 14,113)	Men (*N* = 43,730)	Women (*N* = 22,052)	Total (*N* = 65,782)		
Age at diagnosis (years), mean (SD)	65.3 (14.9)	71.1 (15.1)	67.2 (15.2)	64.4 (14.6)	70.7 (15.0)	66.5 (15.0)		
Level of education, *N* (%)								
≤ 9 years	3560 (37.4)	2169 (47.2)		15,531 (35.5)	9419 (42.7)			
10–12 years	4047 (42.6)	1655 (36.0)		17,349 (39.7)	7699 (34.9)			
> 12 years	1766 (18.6)	690 (15.0)		10,254 (23.5)	4509 (20.5)			
Comorbidities, total (%)	74.4	81.2	76.6	52.8	61.3	55.7		
Comorbidities suggested to increase SU level (%)	52.2	62.8	59.1	25.7	31.7	27.7		
Psoriasis, PR (95% CI)	3.6 (3.3–4.0)	4.0 (3.4–4.6)	3.7 (3.4–4.1)	2.7 (2.5–2.8)	2.8 (2.6–3.0)	2.7 (2.6–2.8)	1.4 (1.3–1.5)	< 0.001
Organ transplantation, PR (95% CI)	1.1 (0.9–1.3)	1.2 (0.9–1.5)	1.1 (1.0–1.3)	0.4 (0.3–0.5)	0.5 (0.4–0.5)	0.4 (0.4–0.5)	2.8 (2.3–3.4)	< 0.001
Renal disease, PR (95% CI)	12.2 (11.5–12.9)	11.1 (10.1–12.1)	11.8 (11.3–12.4)	5.1 (4.9–5.3)	3.4 (3.2–3.6)	4.5 (4.4–4.7)	2.6 (2.5–2.8)	< 0.001
Use of diuretics, PR (95% CI)	39.1 (37.8–40.4)	53.4 (51.3–55.5)	43.7 (42.7–44.8)	15.8 (15.5–16.2)	24.3 (23.6–25.0)	18.7 (18.4–19.0)	2.3 (2.3–2.4)	< 0.001
Obesity, PR (95% CI)	9.5 (8.9–10.1)	12.2 (11.2–13.2)	10.4 (10.0–11.0)	3.5 (3.3–3.7)	4.8 (4.5–5.1)	3.9 (3.8–4.1)	2.6 (2.5–2.8)	< 0.001
Alcoholism	5.1 (4.6–5.5)	1.8 (1.5–2.2)	4.0 (3.7–4.3)	2.9 (2.7–3.1)	0.9 (0.8–1.1)	2.2 (2.1–2.3)	1.8 (1.6–2.0)	< 0.001
Other comorbidities (%)	69.3	76.9	71.8	48.4	57.3	51.3		
Diabetes, PR (95% CI)	14.9 (14.1–15.7)	18.3 (17.1–19.6)	16.0 (15.4–16.7)	9.5 (9.2–9.8)	8.6 (8.2–9.0)	9.2 (9.0–9.4)	1.7 (1.7–1.8)	< 0.001
Hypertension, PR (95% CI)	64.5 (62.9–66.1)	72.3 (69.9–74.8)	67.0 (65.7–68.4)	41.4 (40.8–42.0)	50.2 (49.3–51.1)	44.3 (43.8–44.9)	1.5 (1.5–1.6)	< 0.001
Coronary heart disease, PR (95% CI)	19.5 (18.6–20.4)	18.2 (17.0–19.5)	19.1 (18.4–19.8)	13.7 (13.3–14.0)	11.8 (11.4–12.3)	13.0 (12.8–13.3)	1.5 (1.4–1.5)	< 0.001
Congestive heart failure, PR (95% CI)	16.2 (16.1–17.8)	21.4 (20.1–22.8)	18.4 (17.7–19.1)	6.5 (6.3–6.8)	7.7 (7.3–8.1)	6.9 (6.7–7.1)	2.7 (2.5–2.8)	< 0.001
Atrial fibrillation, PR (95% CI)	18.6 (17.7–19.5)	19.0 (17.8–20.3)	18.7 (18.0–19.5)	7.5 (7.3–7.8)	7.7 (7.3–8.1)	7.6 (7.4–7.8)	2.5 (2.4–2.6)	< 0.001
Stroke, PR (95% CI)	8.3 (7.7–8.9)	9.4 (8.6–10.3)	8.7 (8.2–9.2)	6.9 (6.6–7.1)	7.9 (7.5–8.3)	7.2 (7.0–7.4)	1.2 (1.1–1.3)	< 0.001
Thromboembolism, PR (95% CI)	9.7 (9.1–10.4)	13.6 (12.6–14.7)	11.0 (10.5–11.6)	5.4 (5.2–5.6)	7.3 (7.0–7.7)	6.0 (5.8–6.2)	1.8 (1.7–1.9)	< 0.001
Peripheral vascular disease, PR (95% CI)	4.5 (4.1–5.0)	5.5 (4.8–6.2)	4.8 (4.5–5.2)	2.2 (2.0–2.3)	2.6 (2.4–2.8)	2.3 (2.2–2.4)	2.1 (1.9–2.3)	< 0.001
Chronic obstructive pulmonary disease, PR (95% CI)	4.9 (4.5–5.4)	6.5 (5.8–7.3)	5.5 (5.1–5.9)	2.3 (2.1–2.4)	3.2 (3.0–3.4)	2.6 (2.5–2.7)	2.1 (1.9–2.3)	< 0.001

*CI* confidence interval, *PR* prevalence ratio, *SD* standard deviation, *SU* serum urate

### Prevalence of comorbidities in the study population

#### Comorbidities suggested to increase SU level

At the time of gout diagnosis, 59.1% of gout cases had at least one comorbidity of this category, which was 2.1-fold higher than in controls (27.7%) (Table 1). Exposure to diuretics (43.7%), renal disease (11.8%) and obesity (10.4%) were the most frequent of these comorbidities in gout patients and were 2.3–2.7-fold more common in gout patients at diagnosis vs matched controls (Table 1). The proportions of individuals having undergone organ transplantation and those having diagnosed alcoholism and to a lesser extent psoriasis were also more common in gout patients compared to controls (Table 1).

#### Other comorbidities

At the time of gout diagnosis, the overall proportion of patients having at least one comorbidity of this category was 71.8% in comparison to 51.3% of the controls (Table 1). All cardiovascular diseases were both common and highly associated with gout at the time of gout diagnosis (Table 1), with hypertension being the most frequent in both gout patients (67.0%) and controls (44.3%) (Table 1).

#### Comparison by sex

##### Crude prevalence

At the time of first gout diagnosis, all comorbidities were more frequent in cases compared to controls both overall and stratified by sex (Table 1).

All comorbidities, except for alcoholism, renal disease and CHD, had higher point prevalence in women compared to men, with nonoverlapping 95% CIs for use of diuretics (53.4% vs 39.1%), obesity (12.2% vs 9.5%), diabetes (18.3% vs 14.9%), hypertension (72.3% vs 64.5%), CHF (21.4% vs 16.2%), thromboembolism (13.6% vs 9.7%) and COPD (6.5% vs 4.9%). In men, only diagnosed alcoholism (5.1% vs 1.8%) was significantly more common.

##### Age-standardized prevalence

The higher age of women at diagnosis (71.1 years compared to 65.3 years in men) was taken into account in two separate analyses.

**Fig. 2:**
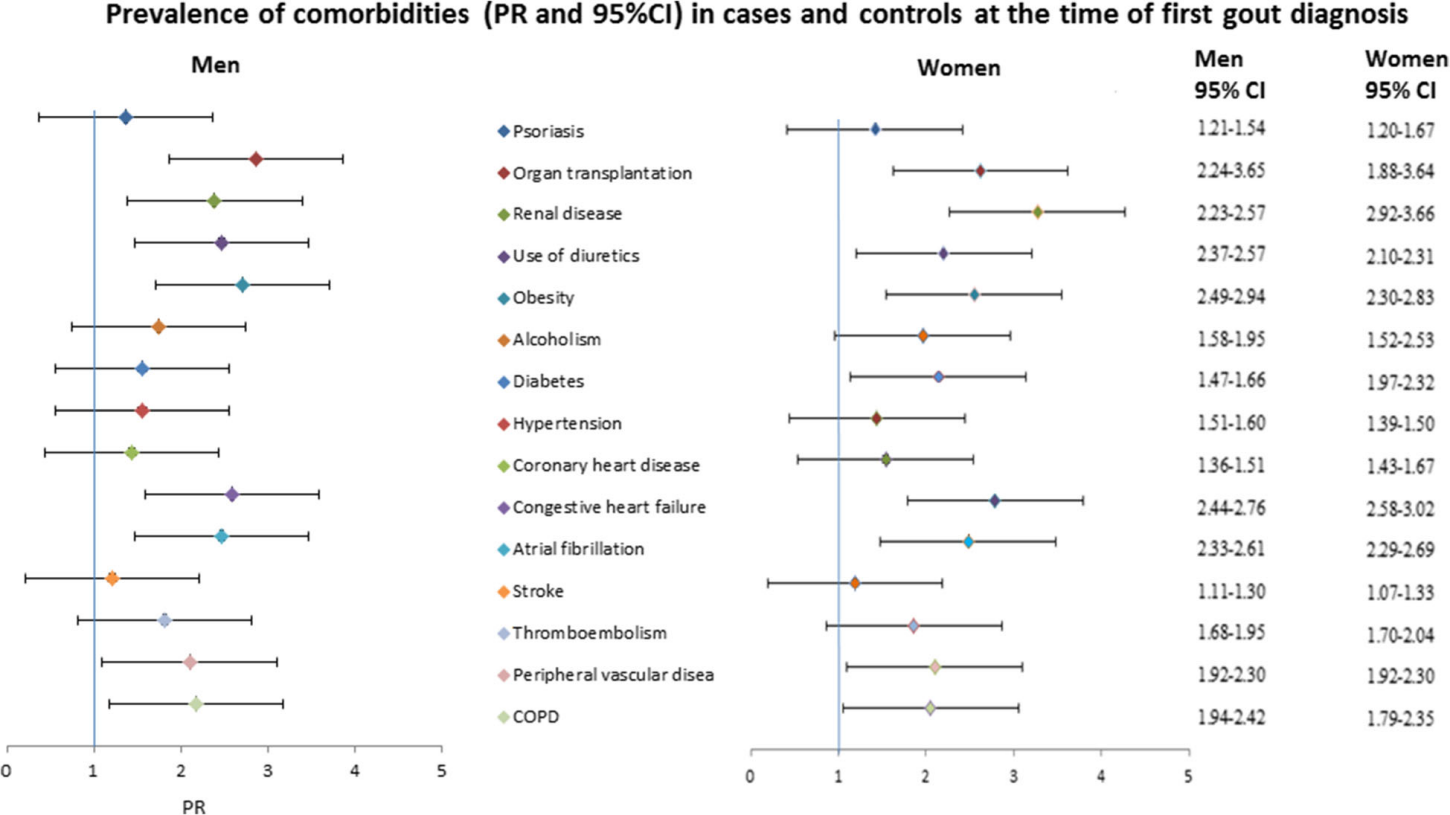
Prevalence rates (95% CIs) of comorbidities in cases and controls for men and women at first gout diagnosis. CI confidence interval, COPD chronic obstructive pulmonary disease, PR prevalence ratio

First, the PRs for comorbidities were calculated by sex for each comorbidity (Fig. 2) compared to age-matched controls. The pattern of PRs was overall similar for men and women, although there were slightly higher PRs (nonoverlapping 95% CIs) for men for diagnosed hypertension and use of diuretics, and for women with regard to diagnosed renal disease and diabetes compared to the opposite sex (Fig. 2).

Second, we compared the age-standardized prevalence of comorbidities in men and women with gout (Table 2). In these analyses, comorbidities suggested to increase SU levels did not differ substantially (overlapping 95% CIs) and were numerically similar in men and women, with the exception of a slightly higher prevalence (but overlapping 95% CIs) for diagnosed obesity and use of diuretics in women.

**Table 2:** Age-standardized prevalence (95% CI) of comorbidities in men and women with gout at the time of first diagnosis

	Gout cases (*N* = 14,113)			
	Men (*N* = 9513), prevalence (%)	95% CI	Women (*N* = 4600), prevalence (%)	95% CI
Comorbidities suggested to increase SU level				
Psoriasis	3.7	2.8–4.5	3.3	2.6–4.1
Organ transplantation	1.3	0.8–1.8	1.8	0.9–2.6
Renal disease	8.2	7.4–9.0	8.3	6.8–9.8
Use of diuretics	21.9	20.9–22.8	24.1	22.7–25.6
Obesity	10.2	8.9–11.6	12.5	10.8–14.3
Alcoholism	5.5	4.7–6.3	3.5	2.1–4.8
Other comorbidities				
Diabetes	8.7	8.1–9.3	9.9	8.9–10.9
Hypertension	40.1	38.6–41.6	39.4	36.9–41.9
Coronary heart disease	9.4	9.0–9.9	6.4	5.8–7.0
Congestive heart failure	7.7	7.3–8.1	6.6	6.0–7.1
Atrial fibrillation	9.0	8.4–9.5	6.0	5.4–6.5
Stroke	4.1	3.7–4.5	3.3	2.8–3.7
Thromboembolism	5.2	4.8–5.6	6.6	5.8–7.5
Peripheral vascular disease	2.1	1.9–2.3	2.1	1.7–2.5
COPD	2.4	2.1–2.6	3.1	2.6–3.5

*CI* confidence interval, *COPD* chronic obstructive pulmonary disease, *SU* serum urate

On the other hand, the age-standardized prevalence for other comorbidities such as CHD, CHF and AF was significantly higher in men (nonoverlapping 95% CIs) compared to that for women, whereas the age-standardized prevalence for thromboembolism and COPD was significantly higher in women (nonoverlapping 95% CIs).

### Sensitivity analyses

To assess the robustness of the results we also calculated the prevalence of the various comorbidities, defining gout by a stricter definition of having had two or more visits to a physician with gout over time. The prevalence of comorbidities when using the stricter case definition was overall similar to that in the main analyses (Additional file 2: Table S2).

## Discussion

In this large, population-based study of patients with gout, recruited from both primary and specialized health care, we found that both comorbidities suggested to increase SU level and other comorbidities were more common in gout cases vs population-based controls, both overall and for men and women separately. All comorbidities, except for alcoholism, were or tended to be more frequent in women than in men at the time of first gout diagnosis. When standardizing for age, these sex differences were attenuated, and diagnoses of CVD were actually more prevalent in men. Since gout may be diagnosed several years after the first attack [50], both hyperuricemia and gout may have been present for a considerable time before and a causal relation to CVD or other comorbidities is not possible to deduce from the present study. Regarding occurrence of comorbidities suggested to increase SU level, there were only minor sex differences after age standardization, which suggests large similarities in the pathophysiologic pathways leading to gout in men and women.

The prevalence of some comorbidities in our study, such as hypertension, CHF, diabetes, PVD and renal disease, was higher than that in the study by Kuo et al. [43], despite the fact that both studies are register based, include a large group of patients and report occurrence of comorbidities at the time of first gout diagnosis. The prevalence of psoriasis, however, was almost the same in both studies. Possible explanations, apart from the different geographical setting, could be that the mean age of gout patients in our study was 5 years higher and that our study had a slightly higher proportion of women (32.6% vs 27.5%), who were found to have higher occurrence of associated comorbidities than men.

Other studies that have examined sex differences in occurrence of comorbidities by sex differ to some extent from ours. One previous study on established gout by Harrold et al. from 2017 [44] showed, in line with the present study, that women had higher prevalence of obesity, hypertension and diabetes mellitus and were more likely to take diuretics compared to men, although this study was based on a smaller sample of patients identified from an American network of rheumatologists. In another previous American study [45] based on 6133 patients diagnosed with gout as part of their health insurance plans, all evaluated comorbidities (obesity was not included) were more frequent in women with gout compared to men. On the other hand, these studies showed that women had higher prevalence of kidney disease compared to men, which is not confirmed in our study. One possible explanation could be that our study is conducted at the time of first diagnosis of gout, whereas the previous studies examined prevalent gout, and that the mean age of our male patients was 5 and 7 years higher respectively than in the two previous studies. There is also one small clinical study by De Souza et al. [47] based on only 58 patients that showed no differences in the occurrence of comorbidities between women and men, which could be explained by the small sample size of the study. We have also shown that thromboembolism and COPD were more frequent in women with gout compared to men. The association between these comorbidities and gout has not been studied before. The higher prevalence in women might suggest some differences in pathophysiologic pathways or risk profile between men and women, but it is not possible to study the underlying mechanisms in the present study.

Regarding CVD, we found a higher occurrence in men compared to women, when adjusting for age. Current literature on risk for cardiovascular events in men and women with gout reports conflicting results. In line with the present study that showed higher prevalence of CHD and CHF in men, when we standardized for age, Krishnan et al. [26] showed that men with hyperuricemia had an increased risk for myocardial infarction in a controlled trial examining the efficacy of risk reduction in men at high risk for vascular events. Results from the Framingham study [25] showed an increased risk for CHD and angina in men with gout, but not in women, independently of other cardiovascular risk factors. Choi and Curhan [4] showed in a large prospective study that men with gout had higher overall mortality compared to men without gout. In contrast, De Vera et al. [51] in a population-based cohort showed higher risk for myocardial infarction in women compared to men, and Clarson et al. [34] in a population representative cohort in the UK showed that female patients with gout were at greater risk for any vascular event compared to men with gout. These discrepancies could be explained by differences between studies in study design, sample size and selection of the study population. Regarding potential explanatory mechanisms for higher frequency of CVD in men, we showed that other conventional risk factors for CVD, such as obesity, hypertension or diabetes, were not higher in men, although smoking and dyslipidemia could not be taken into account due to lack of data.

A previous study by Wright et al. [39] showed that obesity, use of diuretics and, to a lesser extent, renal disease had a significant influence on plasma urate concentrations and allopurinol daily maintenance dose. According to this study, dose requirements to achieve treatment goals for SU were found to increase by 2-fold to over 3-fold with increasing total body weight and were 1.25–2 times higher in those taking diuretics, whereas renal function had a relatively modest impact on the required allopurinol dose. Another study [40] suggests that the effect of decreased eGFR and diuretic use is explained by their ability to increase SU levels and not by any effect on the dose–response relationship between the given allopurinol dose and the change in urate levels. Considering that women in our study and previous studies [44] have a trend toward a higher prevalence of obesity and use of diuretics, factors that increase urate levels in serum, these comorbidities should be considered when initiating and adjusting ULT in women.

Some possible limitations should be acknowledged. The identification of gout patients was based on ICD codes, which may have led to some misclassification bias. However, according to a previous validation study [49], a more strict definition of gout requiring one or more visits with an ICD-coded gout diagnosis by a rheumatologist or two or more visits in primary care was found to have a high positive predictive value for fulfilling the various classification criteria for gout. The comorbidity pattern of gout cases in the present study was very similar to the comorbidity pattern of those fulfilling the stricter case definition (Additional file 2: Table S2), suggesting similar validity for both case definitions. Regarding definition of comorbidities, the validity of ICD codes in the Swedish national patient registry is generally very high [48], although not all diagnoses used in this study have been validated. There is, thus, a possible risk for underestimation of some comorbidities, in particular obesity and alcoholism, when ICD codes are used for definitions. However, there is no reason to believe that such underestimation would be of different magnitude in cases compared to controls and in women compared to men. Smoking, which is a major risk factor for CVD, could not be taken into account because of a lack of data. Finally, the study was performed in the western part of Sweden, which may hamper generalizability. However, according to previous reports, the sociodemographic distribution and healthcare seeking in this region are very similar to Sweden as a whole [52, 53].

This study has also several strengths. First, it is a population-based study, including all gout cases in the region, which reduces the risk of selection bias. Second, patients were identified from both primary and specialized health care, which covers all of the different phenotypes of gout, from mild to severe disease. Third, several unrelated independent data sources were used. For instance, case identification and information regarding medication or education level were retrieved from completely independent sources. Fourth, the study includes a large number of subjects with gout and controls, which generates statistically robust estimates for the occurrence of the various comorbidities.

## Conclusions

This large, population-based study shows that all patients with gout have higher occurrence of many comorbid conditions at the time of first diagnosis, compared to matched controls from the general population. The majority of these comorbidities are more common in women than in men with gout. In particular, the higher occurrence of diuretic use and obesity in women may need to be taken into account when initiating ULT.

## Additional files

Additional file 1: **Table S1.** Definition of comorbidities based on ICD and/or ATC codes (DOCX 94 kb)

Additional file 2: **Table S2.** Demographic characteristics and prevalence (95% CIs) of comorbidities for gout cases and controls, where cases were defined according to the strict definition of requiring ≥ 2 visits with a diagnosis of gout (DOCX 98 kb)

## Abbreviations

AF: Atrial fibrillation
ATC: Anatomical Therapeutic Chemical Classification System
CHD: Coronary heart disease
CHF: Congestive heart failure
CI: Confidence interval
COPD: Chronic obstructive pulmonary disease
CVD: Cardiovascular disease
eGFR: Estimated glomerular filtration rate
ICD: International Statistical Classification of Diseases
LISA: Longitudinal Integration Database for Health Insurance and Labor Market Studies
MSU: Monosodium urate
PDR: Swedish Prescribed Drug Register
PR: Prevalence ratio
PVD: Peripheral vascular disease
SD: Standard deviation
SU: Serum urate
ULT: Urate lowering therapy
VEGA: Western Swedish Health Care Region
