# Correction to: Comorbidity in gout at the time of first diagnosis: sex differences that may have implications for dosing of urate lowering therapy

**DOI:** 10.1186/s13075-018-1700-2

**Published:** 2018-09-10

**Authors:** Panagiota Drivelegka, Valgerdur Sigurdardottir, Anna Svärd, Lennart T. H. Jacobsson, Mats Dehlin

**Affiliations:** 1 Department of Rheumatology and Inflammation Research, Sahlgrenska Academy, University of Gothenburg, Grona Straket 12, Sahlgrenska University Hospital, 413 45 Gothenburg, Sweden; 2 Centre of Clinical Research Dalarna, Falun, Sweden

## Correction

Unfortunately, after publication of this article [1], it was noticed that Fig. 2 is incorrect. The correct Fig. 2 can be seen below.

**Fig. 2:**
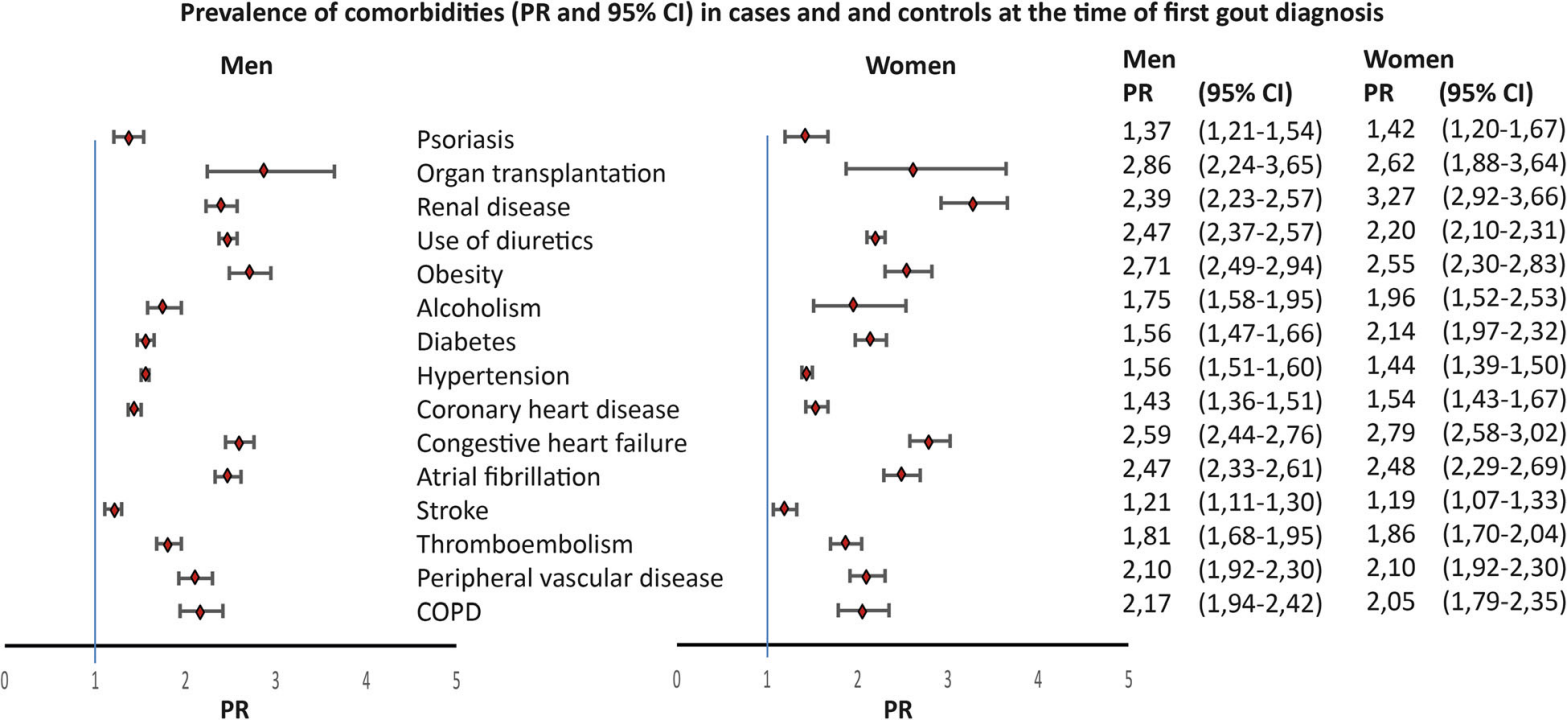
Prevalence rates (95% CIs) of comorbidities in cases and controls for men and women at first gout diagnosis. CI confidence interval, COPD chronic obstructive pulmonary disease, PR prevalence ratio
