# Supplementary material for: Comorbidity in gout at the time of first diagnosis: sex differences that may have implications for dosing of urate lowering therapy
Source: Arthritis Res Ther. 2018 Jun 1;20:108. doi: 10.1186/s13075-018-1596-x (PMC5984404; doi:10.1186/s13075-018-1596-x)
Supplement: Supplementary file 1 — Table S1. Definition of comorbidities based on ICD and/or ATC codes (DOCX 94 kb) [file 13075_2018_1596_MOESM1_ESM.docx]

| **Diagnosis** | **ICD code** | **ATC code** |
| --- | --- | --- |
| Gout | M10, M14.0, M14.1 |  |
| Psoriasis | L40 |  |
| Organ transplantation | Z94 |  |
| Renal disease | N00-N08, N11-22 |  |
| Use of diuretics |  | C03A, C03B, C03C C07BB, C09BA02/03/05/06/08/09/15, C09CA01, C09DA01-04, C09DA06-07, C09XA52/54 |
| Obesity | E66 | A08 |
| Alcoholism | Z72.1, F10 |  |
| Diabetes | E10-14, O24 |  |
| Hypertension | I10-I15 | C02, C07-09 |
| Coronary heart disease (CHD) | I20-I25 |  |
| Congestive heart failure (CHF) | I50 |  |
| Atrial fibrillation | I48 |  |
| Stroke | I60-I64, G45 |  |
| Thromboembolism | I26, I70-I72, I80-I82 |  |
| Peripheral vascular disease (PVD) | I73.9 |  |
| Chronic obstructive pulmonary disease (COPD) | J44 |  |

Table S1: Definition of comorbidities based on ICD- and/or ATC-codes
