# Supplementary material for: Comorbidity in gout at the time of first diagnosis: sex differences that may have implications for dosing of urate lowering therapy
Source: Arthritis Res Ther. 2018 Jun 1;20:108. doi: 10.1186/s13075-018-1596-x (PMC5984404; doi:10.1186/s13075-018-1596-x)
Supplement: Supplementary file 2 — Table S2. Demographic characteristics and prevalence (95% CIs) of comorbidities for gout cases and controls, where cases were defined according to the strict definition of requiring ≥ 2 visits with a diagnosis of gout (DOCX 98 kb) [file 13075_2018_1596_MOESM2_ESM.docx]

|  | **Gout Cases** | | | **Controls** | | |
| --- | --- | --- | --- | --- | --- | --- |
|  | **Men**  (N=2.784)  N (%) | **Women**  (N=971)  N (%) | **Total**  (N=3.755)  N (%) | **Men**  (N=12.852)  N (%) | **Women**  (N=4.658)  N (%) | **Total**  (N=17.510)  N (%) |
| **Mean age at diagnosis, years (SD)** | 64.6 (15.0) | 74.3 (12.8) |  | 63.6 (14.8) | 74.0 (12.8) |  |
| **Level of education** |  | |  |  | |  |
| ≤9 years | 1.032 (37.1) | 518 (53.4) |  | 4.470 (34.8) | 2.166 (46.5) |  |
| 10 -12 years | 1.205 (43.3) | 321 (33.1) |  | 5.120 (39.9) | 1.558 (33.5) |  |
| >12 years | 513 (18.4) | 114 (11.7) |  | 3.088 (24.1) | 836 (18.0) |  |
|  | **Men**  PR (95%CI) | **Women**  PR (95% CI) | **Total**  PR (95%CI) | **Men**  PR (95% CI) | **Women**  PR (95% CI) | **Total**  PR (95%CI) |
| **Comorbidities, total,%** | **73.6** | **92.2** | **78.4** | **51.4** | **64.7** | **54.9** |
| **Comorbidities suggested to increase SU level,%**  TableS2: Demographic characteristics and prevalence (95%CI) of comorbidities for gout cases and controls, where cases were defined according to the strict definition, requiring ≥ 2 visits with a diagnosis of gout. | **52.6** | **75.6** | **58.5** | **24.3** | **32.9** | **26.6** |
| Psoriasis | 3.8  (3.1-4.6) | 4.0  (2.9-5.4) | 3.9  (3.3-4.5) | 2.8  (2.6-3.1) | 2.9  (2.4-3.4) | 2.8  (2.6-3.1) |
| Organ transplantation | 1.1  (0.7-1.5) | 1.5  (0.9-2.5) | 1.2  (0.9-1.6) | 0.4  (0.3-0.5) | 0.6  (0.4-0.8) | 0.4  (0.3-0.5) |
| Renal disease | 11.2  (10.1-12.5) | 12.0  (9.9-14.3) | 11.4  (10.4-12-6) | 4.6  (4.2-5.0) | 2.8  (2.4-3.4) | 4.1  (3.8-4.4) |
| Use of diuretics | 41.1  (38.8-43.5) | 67.5  (62.4-72.8) | 47.9  (45.7-50.2) | 15.3  (14.6-15.9) | 26.2  (24.8-27.7) | 18.2  (17.5-18.8) |
| Obesity | 8.6  (7.5-9.7) | 12.3  (11.1-15.7) | 9.8  (8.8-10.8) | 3.0  (2.7-3.3) | 4.2  (3.7-4.9) | 3.3  (3.1-3.6) |
| Alcoholism | 4.1  (3.4-4.9) | 1.3  (0.8-2.2) | 3.4  (2.8-4.0) | 2.7  (2.4-3.0) | 0.7  (0.5-0.9) | 2.2  (2.0-2.4) |
| **Other comorbidities** | **68.5** | **89.2** | **73.9** | **47.0** | **61.2** | **50.8** |
| Diabetes | 12.5  (11.2-13.8) | 19.7  (17.0-22.6) | 14.3  (13.2-15.6) | 9.5  (9.0-10.1) | 9.0  (8.2-9.9) | 9.4  (8.9-9.8) |
| Hypertension | 65.1  (62.1-68.1) | 85.3  (79.6-91.2) | 70.3  (67.6-73.0) | 40.2  (39.1-41.3) | 53.6  (51.5-55.7) | 43.8  (42.8-44.8) |
| Coronary heart disease (CHD) | 19.6  (18.0-21.3) | 24.3  (21.4-27.6) | 20.8  (19.4-22.3) | 13.3  (12.7-14.0) | 13.1  (12.1-14.1) | 13.2  (12.7-13.8) |
| Congestive heart failure (CHF) | 18.8  (16.5-19.6) | 28.0  (24.8-31.5) | 20.6  (19.2-22.1) | 6.7  (6.3-7.2) | 8.8  (8.0-9.7) | 7.3  (6.9-7.7) |
| Atrial fibrillation | 20.7  (19.0-22.4) | 23.4  (20.5-26.6) | 21.4  (19.9-22.9) | 7.2  (6.8-7.7) | 8.3  (7.5-9.2) | 7.5  (7.1-7.9) |
| Stroke | 7.3  (6.4-8.4) | 11.1  (9.2-13.4) | 8.3  (7.4-9.3) | 6.3  (5.8-6.7) | 8.3  (7.5-9.2) | 6.8  (6.4-7.2) |
| Thromboembolism | 9.5  (8.4-10.7) | 16.1  (13.7-18.7) | 11.2  (10.2-12.3) | 5.2  (4.8-5.6) | 7.9  (7.2-8.8) | 5.9  (5.6-6.3) |
| Peripheral vascular disease (PVD) | 4.7  (3.9-5.5) | 7.4  (5.9-9.3) | 5.4  (4.7-6.2) | 2.2  (2.0-2.5) | 2.9  (2.4-3.4) | 2.4  (2.2-2.6) |
| Chronic obstructive pulmonary disease (COPD) | 4.6  (3.9-5.5) | 7.1  (5.6-8.9) | 5.3  (4.6-6.1) | 2.0  (1.8-2.3) | 3.4  (2.9-3.9) | 2.4  (2.1-2.6) |
